# Using mHealth to Increase Treatment Utilization Among Recently Incarcerated Homeless Adults (Link2Care): Protocol for a Randomized Controlled Trial

**DOI:** 10.2196/resprot.9868

**Published:** 2018-06-05

**Authors:** Jennifer M Reingle Gonzalez, Michael S Businelle, Darla Kendzor, Michele Staton, Carol S North, Michael Swartz

**Affiliations:** ^1^ Department of Epidemiology, Human Genetics and Environmental Sciences University of Texas School of Public Health Dallas, TX United States; ^2^ Department of Family and Preventive Medicine The University of Oklahoma Health Sciences Center and Stephenson Cancer Center Oklahoma City, OK United States; ^3^ Department of Behavioral Science College of Medicine University of Kentucky Lexington, KY United States; ^4^ Metrocare Services and Department of Psychiatry University of Texas Southwestern Medical Center Dallas, TX United States; ^5^ Department of Biostatistics School of Public Health University of Texas Houston, TX United States

**Keywords:** case management, criminal justice, treatment

## Abstract

**Background:**

There is a significant revolving door of incarceration among homeless adults. Homeless adults who receive professional coordination of individualized care (ie, case management) during the period following their release from jail experience fewer mental health and substance use problems, are more likely to obtain stable housing, and are less likely to be reincarcerated. This is because case managers work to meet the various needs of their clients by helping them to overcome barriers to needed services (eg, food, clothing, housing, job training, substance abuse and mental health treatment, medical care, medication, social support, proof of identification, and legal aid). Many barriers (eg, limited transportation, inability to schedule appointments, and limited knowledge of available services) prevent homeless adults who were recently released from incarceration from obtaining available case management, crisis management, substance abuse, and mental health services.

**Objective:**

The aim of the Link2Care study is to assess the effectiveness of a smartphone app for increasing case management and treatment service utilization, and in turn reduce homelessness and rearrest. The goals of this research are to (1) assess the impact of an innovative smartphone app that will prompt and directly link recently incarcerated homeless adults to community-based case management services and resources and (2) utilize in-person and smartphone-based assessments to identify key variables (eg, alcohol or drug use, social support, psychological distress, and quality of life) that predict continued homelessness and rearrest.

**Methods:**

Homeless adults (N=432) who enroll in a shelter-based Homeless Recovery Program after release from the Dallas County Jail will be randomly assigned to one of the three treatment groups: (1) usual case management, (2) usual case management plus smartphone, and (3) usual case management with a study-provided smartphone that is preloaded with an innovative case management app (smartphone-based case management). Those assigned to smartphone-based case management will receive smartphones that prompt (twice weekly) connections to shelter-based case managers. The app will also offer direct links to case managers (available during normal business hours) and crisis interventionists (available 24 hours a day, 7 days a week) with the touch of a button.

**Results:**

Recruitment began in the spring of 2018, and data collection will conclude in 2021.

**Conclusions:**

This research represents an important step toward integrated service connection and health care service provision for one of the most underserved, high need, and understudied populations in the United States.

**Trial Registration:**

ClinicalTrials.gov NCT03399500; https://clinicaltrials.gov/ct2/show/NCT03399500 (Archived by WebCite at http://www.webcitation.org/6zSJwdgUS)

**Registered Report Identifier:**

RR1-10.2196/9868

## Introduction

### Background

An estimated 3.5 million people experience homelessness each year in the United States [1], and 6.2% of US adults have been homeless at some point in their lifetime [2]. Homeless adults are more likely than domiciled adults to be male, single, African American [3-7], have very low income, and have average life expectancies that are 8 (women) to 13 (men) years shorter [8]. Homeless adults are more likely than domiciled adults to spend time in jail [9], and as many as 32% of jailed adults report being homeless in the year before their arrest [3,9]. Furthermore, homeless adults are more likely to return to jail after incarceration than domiciled adults [10]. In Texas, more than half of adults released from county jails are rearrested within 1 year [11], and many of those rearrested are homeless [12]. In Dallas County alone, 5530 homeless adults were incarcerated in 2013 at an estimated cost of US$12,557,406 (calculated in 2014) [13].

Incarcerated homeless adults have a variety of risk factors that increase the likelihood of rearrest. For instance, homeless inmates are more likely than domiciled inmates to have histories of mental illness or substance use disorders [9,12]. The research team’s preliminary studies indicated that homeless adults released from jail in the past year were more likely than those not recently incarcerated to have a history of substance use or mental health problems [14]. Thus, there is a strong need for mental health and substance abuse treatment among homeless adults following their release from jail. Studies have indicated that case management services for substance use and psychological distress can attenuate the link between homelessness and incarceration [15-18]. The overall significance and scope of this issue was eloquently stated by Kushel and colleagues in their evaluation of the *revolving door* of homeless incarceration [16]: “High rates of imprisonment among homeless populations may be the end result of a system that does not provide access to timely services, including access to housing, health care, mental health care, and substance abuse treatment, and systems that have obstacles preventing receipt of these services by people exiting prison.” Thus, individuals who leave jail and return to the community without stable housing are at increased risk for premature mortality [19,20] and rearrest [10,12] and are critically in need of interventions that increase access to services [15].

### Case Management

Case management is the professional coordination of individualized care [21]. Specifically, case managers link individuals with relevant services and help them to overcome barriers to service utilization. In addition, case managers engage in client assessment, practical support, service planning, advocacy, and monitoring of service utilization and progress [17,22,23]. More intensive case management services (often employed with homeless adults) include a multidimensional approach with integrated counseling, independent living skills building, assertive outreach, and crisis intervention [24]. Case management has been shown to be effective in improving housing stability, mental health, quality of life (QoL), and social functioning, while reducing substance use, hospitalization stays, and incarceration in at-risk populations [15,25-27], including homeless and recently incarcerated adult populations (see meta-analysis [23]).

Homeless individuals have many needs following release from incarceration, including housing, employment, substance abuse and mental health treatment, medical care, medication, social support, proof of identification, and legal aid [3,9,28,29]. Although many existing public services address these needs, there are many barriers to service utilization and obtaining stable housing [30]. For example, it is difficult for an individual to identify which services and housing placement programs are and are not available to those with histories of arrest, substance abuse, and serious mental illness [15,31-33]. Furthermore, inability to provide valid identification (eg, driver’s license or birth certificate) limits the ability to obtain employment assistance and disability services and is often a rationale for arrest by police [30]. In addition, lack of access to transportation reduces the ability of this population to access free and available community services (eg, food, clothing, temporary housing, and obtaining identification) [34]. There are also many specific barriers to the utilization of case management among homeless adults, including lack of a permanent address, telephone service, transportation to case management visits, and access to service providers’ contact information [8,35-39]. These factors reduce the ability of homeless adults to schedule appointments and limit the ability of providers to contact patients regarding appointments [40,41].

### Smartphone Use Among Homeless Adults

Cell phone ownership is common among homeless adults, with 58.4% reporting that they had active cell phone service in 2014 [42], which is not surprising because there are government programs that pay for cell phone service for qualifying very low income adults [43]. Furthermore, findings from other research suggest that 71.9% of homeless adults in Oklahoma City had an active cell phone or smartphone in 2016 (56.1% had an active smartphone, unpublished data [44]). Other studies have indicated that 70% of homeless adults who have cell phones use them to connect with peers and family members, 32% carry a phone for safety reasons (eg, access to emergency services), and 23% use a phone to communicate with current or potential employers [39-41]. Although 62% of homeless youths possessed activated cell phones, only 17% were using their cell phone to connect to case managers [40]. Thus, initial evidence indicates that cell phones are already being widely used in homeless populations, but few homeless adults are using their phones to contact case managers who have the primary role of linking individuals to care and coordinating care for those in need. Thus, a significant opportunity for novel interventions is being missed. Smartphone apps may be a novel way to facilitate direct access to case management and may be a practical and affordable means by which to reduce barriers to service utilization in vulnerable and hard-to-reach populations. In our recent studies that have used smartphones, the cost for an activated smartphone with monthly talk, text, and internet has been under US $20 per month, which is equivalent to less than the cost of one-third of 1 day in the Dallas County Jail [45].

Aside from demographic variables and history of mental illness or substance use or abuse, very few predictors of rearrest and sustained homelessness have been identified [9]. To date, all studies that have examined predictors of incarceration, rearrest, health, and continued homelessness among homeless adults have used traditional in-person assessment methods that are usually conducted retrospectively or months or years before the predicted outcome [9,16,37,46-49]. Studies have indicated that traditional assessment methodologies provide biased and inaccurate estimates because of recall bias and errors in memory (eg, assessing the number of drinks consumed or level of depression or anxiety over the past week or month) [50,51]. Ecological momentary assessment (EMA), in which handheld devices are used to capture “real time” experiences that vary daily (or from moment to moment), is currently the most accurate way to measure phenomena in real time in natural settings [50,52]. Although EMA has been used in a variety of populations and with multiple health outcomes, only 1 study [53] outside of our own work [54] has collected EMA data in homeless adults. The current study (Link2Care) will identify key variables, measured proximally (EMA data) and distally (traditional in-person assessments and EMAs), that predict alcohol and drug use, social support, psychological distress, and QoL. These rich data will address knowledge gaps that have limited our understanding of and ability to intervene in this marginalized population.

**Figure 1:**
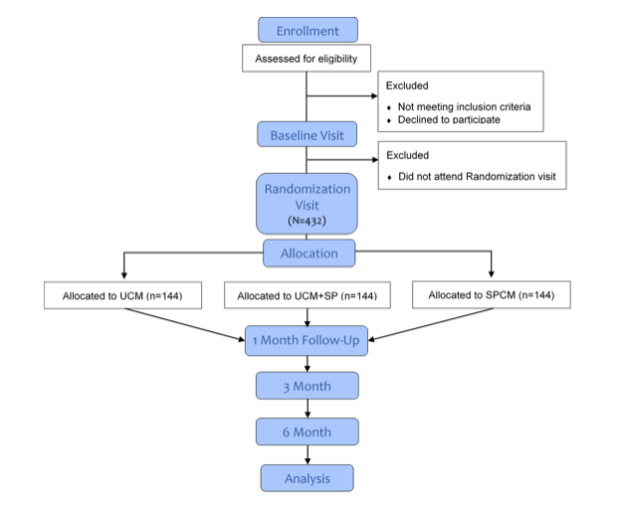
CONSORT flow diagram for Link2Care randomized controlled trial. UCM: usual case management; SP: smartphone; SPCM: smartphone-based case management.

In the Link2Care three-arm randomized controlled trial (RCT), homeless adults who enroll in a shelter-based Homeless Recovery Program following release from the Dallas County Jail (N=432) will be randomized to one of three conditions: usual case management (UCM), UCM plus smartphone, or UCM plus smartphone-based case management (SPCM). SPCM will be delivered through the Insight mHealth platform. Insight is a versatile mobile app platform that enables researchers to rapidly create and schedule smartphone-based assessments and interventions [55]. The app will not provide case management and crisis intervention services directly; rather, it will prompt twice weekly contact with their case manager and provide links to service providers through the touch of a button. Specifically, we will compare case management and crisis management service utilization among recently incarcerated homeless adults who are randomized to the UCM, UCM plus smartphone, and SPCM conditions. We will also estimate the effect of treatment condition on alcohol use, drug use, and psychological distress, and identify key factors (alcohol and drug use, social support, psychological distress, QoL) that predict rearrest and nights spent homeless using traditional and smartphone-based assessment approaches. A flowchart of the procedures is provided in Figure 1.

## Methods

### Setting and Procedure

Link2Care is an unblinded RCT. Participants (N=432) will be recruited at a large homeless shelter in Dallas, Texas. The shelter has 80 employees and on-site partners that provide services (eg, meals, mental health and substance abuse counseling, case management, housing placement, and job readiness training) to approximately 85% of all homeless adults in Dallas County each year. The shelter conducts approximately 366 new intakes each month, and approximately half of all new intakes enroll in the optional Homeless Recovery Program. Overall, the shelter provides services to 2847 unique homeless adults each month.

### Eligibility Criteria

Textbox 1 shows the eligibility criteria for interested individuals.

### Participant Recruitment

Individuals who identify as homeless upon release from the Dallas County Jail will be given a two-sided flier by jail reentry staff. One side of the flier will provide information about services that may be useful to homeless adults in Dallas (eg, directions to the shelter and other nearby shelters where they may obtain meals, shelter, housing assistance, and other services). The other side of this flier will be used to briefly describe this study. Each flyer will have a unique identification number to allow the researchers to track the response rate based upon the number of flyers distributed. This flyer will be considered a “ticket” for screening and potential participation into this study.

Individuals who were released from the Dallas County Jail in the past month and present at the shelter will complete a shelter intake form and enroll in the shelter’s Homeless Recovery Program (this is the current standard of care at the shelter). These individuals will receive information about this study and will be informed that shelter services are not contingent upon study enrollment. Eligible adults who remain interested the study will be directed by the shelter intake coordinator to the study research staff for screening.

Those who meet study inclusion criteria and provide informed consent will complete the baseline assessment measures and will be given an appointment to return to the shelter within 72 hours for randomization into one of three study conditions. Participants will return to the shelter for follow-up assessments 1, 3, and 6 months after the randomization visit. All participants, regardless of condition, will be compensated for completing each in-person visit.

During the informed consent process, a member of the research team will explain to all participants that no information that they provide during the study will be shared with the Dallas County Jail. The research team will also discuss our Certificate of Confidentiality with each prospective participant and how the Certificate will be used to refuse requests to disclose information from all outside organizations, including Dallas County.

### Randomization Plan

We will use permutated-block randomization to avoid the disadvantage of simple randomization where treatment imbalance can occur periodically. We will use a block size of 12 to ensure that an equal number of 4 subjects are randomized into each arm within an individual block. On the basis of our total sample size of 432, we will perform permutated-block randomization for a total of 36 blocks.

### Study Conditions

#### Usual Case Management Group

The UCM group will receive the standard Homeless Recovery Program currently offered at the shelter. To qualify for the standard Homeless Recovery Program, individuals must complete a shelter intake and substantiate homelessness (eg, provide evidence that they spent the previous night in a shelter or jail). The shelter intake includes a comprehensive needs assessment, and demographic information is obtained. Following intake, shelter guests receive a day pass that grants them access to many of the services available at the shelter (eg, meals, showers, laundry, phone, mail, library, barber shop, and storage space for their belongings). Those who enroll in the shelter’s Homeless Recovery Program receive an identification card and can gain access to additional services including case management, onsite mental health and substance abuse counseling, housing assistance, disability or veterans benefits assistance, job readiness training, legal aid, and bus passes. Although these services are freely available to all guests enrolled in the Homeless Recovery Program, many services are offered only during normal business hours, and in-person visits are the norm.

Shelter case managers are licensed professional counselors or Master’s level clinicians who adhere to the Standard Case Management Model [23,58]. Case managers assist homeless adults with (1) Developing care and housing plans, (2) Making and maintaining linkages to on- and off-site service providers (eg, mental health and substance abuse treatment providers), (3) Obtaining vital documents needed for housing and income (eg, birth certificates, state identification, and social security cards), (4) Job readiness training and placement (if appropriate), (5) Overcoming barriers related to criminal history, (6) Development of and reconnection with support systems, and (7) Transitioning from homelessness to appropriate housing. Case managers also advocate on behalf of homeless adults by serving as a connection between all agencies that will be assisting the guest, their families, and any other involved parties. Guests are encouraged to meet with their case managers weekly; however, shelter data have indicated that those who enroll in the shelter’s Homeless Recovery Program complete a total of 1.95 and 3.12 case management sessions, on average, in the first 1 and 6 months of enrollment, respectively.

Shelter intake specialists are primarily responsible for completing the shelter intake process with shelter guests, determining eligibility for the shelter’s Homeless Recovery Program and ensuring that guests are linked with onsite case management staff and the on or off-site service providers they need (eg, mental health and substance abuse programs). The intake process includes collection of information on behavioral and mental health and treatment history, substance abuse and treatment history, risk and safety assessment, medical history, criminal history, history of homelessness, and assessment of social support and other protective factors. Intake specialists are available to meet with guests at the shelter and over the phone.

**Eligibility criteria.**

Interested participants are eligible for the study if they

- were released from Dallas County Jail in the past month
- plan to reside in the Dallas area for the next year
- enroll in the shelter’s Homeless Recovery Program
- are willing and able to attend the baseline visit, randomization visit, and the 1-, 3-, and 6-month follow-up visits
- score ≥4 on the Rapid Estimate of Adult Literacy in Medicine-Short Form (REALM-SF) [56], indicating >6th grade English literacy level (ie, a 7th grade reading level is necessary to complete assessments; <1% of shelter guests are non-English speakers)
- score >24 on the Mini-Mental State Exam [57], indicating no substantial cognitive impairment

**Table 1:** Smartphone-based case management (SPCM) group smartphone app features.

Feature or button	Description of feature
Call My Care Manager	Clicking this button will automatically call the participant’s assigned case manager. Individual case managers are assigned to all Homeless Recovery Program enrollees, and they are available from 8:00 AM to 5:00 PM Monday to Friday.
Call Crisis Line	Clicking this button will call a representative from a Dallas-based crisis line available 24 hours a day, 7 days a week to help homeless individuals address and overcome crises.
Helpful Websites	Clicking this option will lead to a menu of websites that may be useful to participants (eg, Dallas public transit routes and support group schedules or locations [eg, Narcotics and Alcoholics Anonymous]).
Call Study Staff	Clicking this option will connect participants to study staff if they encounter problems with the study phone or rescheduling missed follow-up appointments.
Payment	This button indicates the amount of incentives that participants have earned for completing ecological momentary assessments to date. These payments will be awarded when each participant presents at the shelter to complete their 1-, 3- and 6-month follow-up assessments.

#### Usual Case Management + Smartphone Group

**Figure 2:**
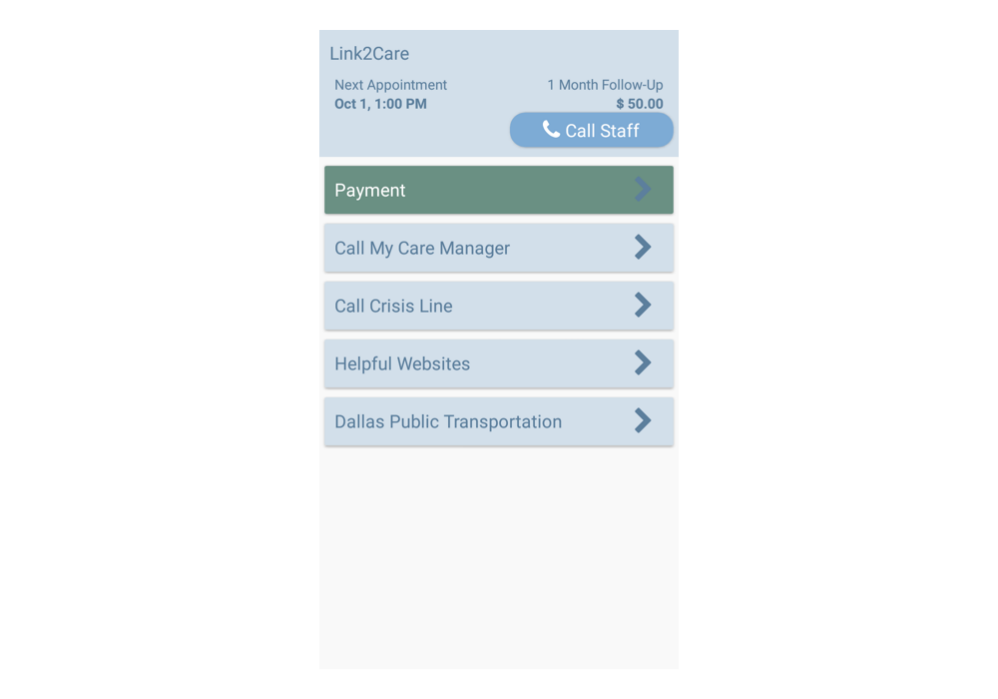
Smartphone-based case management app.

The UCM plus smartphone group will receive UCM and an activated study smartphone (described below), even if they own a personal cell phone. This smartphone only group (without the SPCM app) is necessary to differentiate the effect of the innovative app from provision of a smartphone only. Homeless adults who have phones and access to the internet may have higher levels of social support, which may be related to mental health, QoL, and ability to obtain housing and avoid rearrest [8,41,59]. All smartphones will include standard cellular service that includes unlimited SMS text messaging (short message service), talk minutes, and internet access (speeds are throttled after monthly download limit is reached). Participants will be informed that they may use the phone to make calls, text, and use the internet as they wish during the 6-month course of the study. Participants randomized to the UCM plus smartphone condition will receive phones with a very basic app that will include only the “Call Study Staff” and “Payment” functions (see Table 1 and Figure 2) on the app home screen and will prompt daily EMAs (see EMA description below). Links to case management resources will not be loaded onto phones for participants in UCM plus smartphone condition.

#### Smartphone-Based Case Management Group

Participants who are assigned to SPCM will have access to UCM and will receive a smartphone that is preloaded with an app that will provide direct links to services. Participants will be asked not to discuss SPCM app features with other participants. Smartphones and service plans will be identical to what is provided to the UCM plus smartphone group. SPCM and UCM plus smartphone condition participants will keep the phones at the end of the study.

Recent research has indicated that phone prompts can increase service utilization [60,61]. For example, Lucht showed that twice weekly phone prompts increased phone-based counseling sessions in alcohol dependent patients [60]. To increase the likelihood that SPCM group participants will use the resources available through the app, the phone will be programmed to automatically prompt or suggest a connection with their case manager twice per week. Specifically, the phone will ring or vibrate on two occasions each week at random times between 9:00 AM and 5:00 PM, Monday to Friday, to ask participants if they would like to contact their case manager. Participants will be able to select “No” (this will decline the connection) or “Yes” (this will automatically call their case manager). Participants will be instructed to leave a voice message or speak with an alternate case manager when their case manager cannot be reached. We decided against more frequent prompts to connect with case managers (eg, daily) because of higher participant burden and concern for overwhelming the case management system.

Participants who are randomly assigned to the UCM plus smartphone or SPCM conditions will receive a smartphone at the randomization visit, and they will be asked to carry it with them at all times for 6 months (26 weeks). Date, time, and duration of SPCM app feature use (eg, case manager calls) will be recorded by the app for future analysis. See Table 2 for a summary of study conditions.

### Measures

#### Traditional Measures (In-Person)

Traditional assessment data will be primarily collected on laptop or tablet computers using Questionnaire Development System (QDS) software at in-person baseline and follow-up visits. QDS utilizes a computer-administered self-interview format (ie, *audio computer-assisted self-interviewing*) that reduces data entry errors and the need to retain paper copies of raw data. Each item appears on the computer screen while the program simultaneously reads the item (participants may select their responses only after QDS reads each item). Participants wear headphones so that others do not hear the survey items. Participants have reported few problems using the QDS program, including those with no computer experience. Staff will be available to help participants who may have difficulty. The amount of time needed to complete the QDS-administered questionnaires varies by study visit. On the basis of our previous experience with collecting data in homeless adults, we estimate that the baseline visit will require approximately 1.5 hours to complete, and follow-up assessment visits will require approximately 1 hour.

**Table 2:** Summary of study conditions.

Participants receive	UCM^a^	UCM + plus smartphone	SPCM^b^
Standard intake with service referrals	✓	✓	✓
Standard Homeless Recovery Program	✓	✓	✓
Shelter care managers and crisis management	✓	✓	✓
Provided study smartphone		✓	✓
Provided study smartphone with care linkage app and prompts			✓

^a^UCM: usual case management.

^b^SPCM: smartphone-based case management.

Traditional measures are listed in Textbox 2. Textbox 3 includes numerous constructs that are hypothesized to directly and indirectly (ie, mediation of the treatment effect) affect the study outcomes. In addition, barriers to phone-based case management sessions and staff and participant perceptions of the SPCM app will be assessed. Finally, participants will be asked if they handled or were aware of other participants’ study phones to assess potential cross contamination between study arms. Rearrest and number of homeless nights will be collected using a Timeline Follow-back procedure at all in-person study visits. In addition, arrest data from the Dallas County Jail will be examined to identify participants who are rearrested within 12 months of the randomization visit. This will provide an objective measure of the date and time of arrest, as well as a description of the charges.

#### Ecological Momentary Assessment Measures (Phone-Based)

EMA is currently the most accurate way to measure phenomena in near real time in natural settings [50,52]. Thus, EMA methodology will enable the identification of key variables that predict study outcomes with less bias than traditional in-person assessments. At the randomization visit, those assigned to the UCM plus smartphone and SPCM conditions will be trained on how to use the smartphone to complete EMAs and how to use the “Call Staff” and “Payment” button or options. Those assigned to the SPCM condition will be trained to use the features of the full smartphone app. All participants who receive smartphones will be prompted by the phone to complete one EMA 30 min after waking each day for 6 months beginning on the day of the randomization visit. EMA data, collected over a 6-month period, will be used to identify factors that significantly contribute to alcohol or drug use, QoL, social support, distress, rearrest (ie, because rearrest rates tend to peak within 6-12 months of jail discharge [11]), and continued homelessness (homeless episode duration peaks at 180-190 days [76,77]).

#### Hardware

Participants will use Samsung Galaxy Core Prime smartphones (or equivalent) to complete EMAs. The phone has a 4.5 inch (480x800 pixel resolution) touch screen display, a built-in microphone, earphone jack, speaker, and a rechargeable battery with 13 hours of talk time. It is Wi-Fi and Global Positioning System capable. Participants will navigate through the EMA program and enter data simply by touching the screen. Thus, computer or typing skills are not required. Participants have the ability to call (eg, if they have problems completing EMAs) and receive calls from research staff through the smartphone free of charge.

#### Programming

The mHealth Shared Resource at the University of Oklahoma Health Sciences Center and Stephenson Cancer Center will provide the programming services for the proposed project [55]. The mHealth Shared Resource specifically offers resources that empower researchers to build, test, and launch technology-based assessment and intervention tools. Apps are developed using state-of-the-art cross-platform (eg, Android and Apple) design tools. The mHealth resource employs a program manager and four mobile app programmers who develop and maintain Web and mobile apps and relational databases.

#### Ecological Momentary Assessments

The EMA methodology that will be used in this study is similar to that developed by Shiffman and colleagues [51,78,79] and was used in previous studies conducted by the investigative team [80-84]. EMA items will assess numerous constructs that are hypothesized to be related to the study outcomes (see Textbox 3). The phone will audibly and visually cue EMAs for 5 min and 30 min after each participant’s preset waking time. If the participant does not respond to the initial EMA prompt, the EMA will be recorded as missed, and another prompt will be pushed 1 hour later (this will reduce the likelihood of missed EMAs). On average, EMAs are expected to take 3 to 4 min to complete. All EMAs will be date-, time-, and geolocation-stamped for future analyses. A Certificate of Confidentiality has been obtained from the National Institutes of Health to protect participant data from subpoena.

**Example in-person measures.**

In-person measures

1. Demographics or background
  - Demographics and Homelessness Questionnaire
  - Arrest history [62]
2. Health, mental health, and health behavior
  - Patient Health Questionnaire (depression or anxiety) [63]
  - Mental Health Component from the 12-item Short Form Survey (SF-12) [64]
  - Health-related quality of life [65]
  - Alcohol and drug timeline follow back [66]
3. Stress or stress measures
  - Discrimination [67]
  - Urban Life Stressors Scale [68]
  - Personal victimization [69]
  - Perceived Stress Scale- Short Version [70]
4. Negative affect
  - Aggression [71]
  - Center for Epidemiological Studies-Depression (CES-D) [72]
5. Interpersonal or intrapersonal resources
  - Interpersonal Support Evaluation List-12 [73]
  - Lubben Social Network Scale-6 [74]
6. Homeless Nights Timeline Follow Back [75]
7. Treatment Quality and Satisfaction Survey

**Ecological momentary assessment (EMA) items.**

1. Daily items
  - Positive and negative affect
  - Sleeping arrangements
  - Social support and interactions
  - Stressors
  - Discrimination
  - Prescription medication use
  - Alcohol consumption
  - Other illicit substance use
  - Meal consumption
2. Weekly: Monday assessments
  - Arrest
  - Employment
  - Exposure to crime or violence
  - Emergency room visits
  - Hospitalization
  - Quality of life

#### Smartphone Training

We have developed and successfully implemented a brief user-friendly training protocol for those with limited or no experience using smartphones. Participants will receive hands-on training on study phone use and will watch a brief step-by-step video tutorial (created by the researchers) that demonstrates use of the study smartphone and app features. This video is preloaded onto the home screen of each study phone so that participants may watch and rewatch it at any time. The investigators have achieved high EMA compliance rates (ie, 90.6%, 802/885) of morning EMAs completed) using this protocol in a sample of homeless adults [85].

#### Compensation

Participants will receive compensation for completing each in-person visit (ie, visits 1 and 2=US $30; visits 3-5=US $50) in the form of gift cards. Participants who receive study phones will also be compensated based upon the percentage of EMAs completed since their last in person visit. At the 1-month follow-up visit, participants who completed >90% of daily EMAs will receive a US $50 gift card, those who completed 75% to 89% of EMAs will receive a US $30 gift card, and those who completed 50% to 74% of EMAs will receive a US $20 gift card. Thus, participants may receive up to US $50 for completing EMAs at the 1-month follow-up visit, US $100 at the 3-month follow-up visit (2 months of EMA), and US $150 at the 6-month follow-up visit (3 months of EMA).

#### Data Loss Prevention

To overcome potential loss of data if participants lose the study phone, phones will be programmed to connect to the secure server each day to upload encrypted data. This will ensure that no collected EMA data are lost. This tactic will also allow the researchers to remotely monitor each participant’s EMA completion rate and intervene (eg, call the participant) when this rate is low. Importantly, EMA data will be password-protected and encrypted on the study phone, and only encrypted data are transmitted to the secure server. Thus, study data are only accessible by the research team. If a phone is lost or damaged, it will be remotely cleared of data, and only one replacement phone will be provided to each participant.

#### Participant Emergencies

Participants in all three conditions will be informed that they should utilize the Bridge Homeless Recovery Program or call 911 to manage mental health issues and crises. In addition, participants who are assigned to the SPCM group will be informed that they can click the “Call Crisis Line” button to obtain further assistance in crisis situations. If the participant expresses suicidal plans, symptoms of major depression, panic attacks, acute withdrawal symptoms, etc, during interactions with research staff at scheduled study visits at the Bridge shelter, staff will facilitate immediate connection with Bridge case managers.

### Sample Size

The number of participants (n=144 in each group) was estimated based on the following assumptions: (1) random allocation of participants between three conditions, (2) type I error rate set to 0.05, (3) a 30% dropout rate for each condition [27], (4) targeted minimum power of 0.9, and (5) a conservative increase of 4.5 case management sessions between the UCM and SPCM conditions across the 6-month study period. The estimated increase in case management sessions is based on a previous study [60] showing that 20% of all phone-based prompts to connect with an alcohol treatment counselor resulted in actual treatment sessions.

### Statistical Analysis

Primary analyses will model counts of the total number of case and crisis management sessions that occurred between the randomization visit and the 6-month follow-up across the three conditions using linear regression, with indicator variables to compare the effect of each study group, adjusting for controlled covariates (race or sex or age). We will also perform stratified modeling to determine if the intervention has similar effects across races, sexes, and age. We will adjust for multiple comparisons using the false discovery rate adjustment [86]. For all statistical analyses, the necessary assumptions will be tested before modeling. Remedial measures include variable transformation or generalized linear modeling (such as Poisson regression).

Multilevel models, also known as mixed models, will assess the effect of condition on alcohol and drug use and psychological distress. Covariates for analyses will include baseline characteristics that are known predictors of each outcome, including age, race or ethnicity, employment status, criminal history, and periods of lifetime homelessness. We will also test for interactions between treatment and key demographic variables (eg, race, ethnicity, sex, and age).

Logistic regression of traditional in-person assessments (eg, substance use, social support, psychological functioning, and QoL; see Textbox 3 for other key constructs) and summarized EMA data (eg, affect, stress, discrimination, and alcohol and drug use; see Textbox 3) will be conducted to identify significant demographic, psychosocial, environmental, and behavioral predictors of rearrest in the 12 months following the randomization visit (rearrest status is a binary outcome). Covariates may include treatment group, age, sex, race or ethnicity, education, type of crime, and other variables as appropriate. Change scores (eg, change in social support from baseline to the 1-month follow-up visit) will also be examined as potential predictors of rearrest. If little variation in rearrest status is detected, supplementary survival analyses may be conducted to identify predictors of time to rearrest.

Generalized linear mixed model (GLMM) regression analyses will be used to examine the longitudinal effect of key risk and protective factors on number of homeless nights (measured repeatedly using a timeline follow-back procedure at in-person follow-up visits). GLMM can handle fixed and random effect model parameters, nested designs, and repeated measures with various correlation structures [87,88]. GLMM can also handle different variance functions, unbalanced designs where the number of repeated observations varies across individuals, and the situation where assessments within a week are more highly correlated than assessments separated by multiple weeks or months. We will assess the best way to model the correlation of the repeated measures using the methods of Wolfinger [89] and statistics such as Akaike’s and Schwarz’s information criteria. Adjustments for multiple comparisons will be made according to Westfall and Young [90].

GLMM will also be used to identify proximal predictors of homeless nights (assessed each day) using EMA data. EMAs generate an enormous amount of data; therefore, we will be able to address multiple within- and between-subject questions. For example, key EMA variables (eg, negative affect and stress) and parameters (eg, intercept, slope, quadratic term, and volatility) will be examined as potential predictors of homeless nights. This invaluable information may be used to detect high-risk situations that may be targeted in future “just-in-time adaptive interventions.” EMA data will also allow us to address other important exploratory questions such as (1) What psychosocial changes occur as an individual moves from homelessness into housing and (2) What effect do events such as exposure to discrimination, violence, or hospitalization have on homeless nights and reincarceration.

Finally, the PROCESS macro for SPSS or SAS (described in Hayes [91] and available online [92]) will be used to conduct exploratory mediation analyses to identify variables that mediate the relation between condition and homeless nights and rearrest outcomes. This method uses an ordinary least squares path analytic framework to estimate direct and indirect effects in single and multiple mediation models with bootstrapped CIs. The macro can also be used to evaluate moderated mediation models, including those with dichotomous outcomes (eg, arrest vs no arrest).

## Results

Two separate institutional review boards (IRBs), the Committee for the Protection of Human Subjects at the University of Texas School of Public Health (IRB approval HSC-SPH-15-0632) and the University of Oklahoma Health Sciences Center (IRB approval 8525), have approved the protocol as presented in this manuscript. The smartphone app has been developed (see Figure 2 for a screenshot of the SPCM app home screen), and data collection began in April 2018. Participants will be enrolled for 6 months, and rearrest data will be collected over a 12-month period.

## Discussion

### Research Goals and Hypotheses

Link2Care will be the first study to use smartphones to increase case management sessions among homeless adults. If effective, smartphone apps that remove or attenuate barriers to case and crisis management services could be easily incorporated into other “real world” settings to reduce health disparities among homeless adults. Specifically, we hypothesize that recently incarcerated homeless adults assigned to the SPCM condition will utilize more case and crisis management services than those assigned to UCM or UCM plus smartphone condition. Our study will also compare the effect of treatment condition on alcohol use, drug use, and psychological distress, and we expect that the SPCM group will demonstrate greater improvements in each outcome compared with UCM plus smartphone or UCM. Finally, we will identify key factors (alcohol and drug use, social support, psychological distress, QoL) that predict rearrest and nights spent homeless using traditional and smartphone-based assessment approaches. We hypothesize that key variables that are measured in-person (eg, alcohol or drug use, perceived social support, psychological distress, and QoL) and via daily phone-based assessments (eg, affect, stress, discrimination, and alcohol or drug use) will have direct effects on rearrest and number of homeless nights. These key variables are also hypothesized to mediate the relation between treatment condition and number of homeless nights and rearrest.

We expect that Link2Care will have an important and sustained impact by (1) providing evidence of the utility and effectiveness of an innovative, low cost, highly disseminable, and sustainable smartphone app that links a vulnerable population to freely available services and (2) identifying key mechanisms of treatment that may become intervention targets in future research. It is also conceivable that the SPCM app may reduce victimization, as the overlap between victimization and offending is well-documented [93], especially among those with mental health problems [94]. If effective, efforts will be made to disseminate the app to criminal justice agencies and shelters nationwide.

### Potential Problems and Alternate Strategies

We expect follow-up rates that align with those attained in our previous studies (eg, in one of our previous studies with a similar homeless population, 96% of all participants attended the 1-week and 2-week follow-up visits [when they were carrying the smartphone], and 79% attended the 5-week follow-up visit) [86]. In Link2Care, two-thirds of all participants will be reachable through study phones, and we anticipate high follow-up rates for these participants. We have made efforts to ensure high follow-up rates for those assigned to UCM (they do not receive study phones). Specifically, participants will be asked to provide detailed contact information [95]. These forms have been used to maintain contact with 78% to 88% of recently incarcerated or homeless adults for up to 12 months after enrollment [64,96-98]. In addition, a shelter case manager will assist with contacting participants whom research staff are unable to contact directly using the participant contact form [95,97]. It is important to note that many homeless adults have mailboxes at the shelter, and their forwarding address is obtained when they obtain housing. If participants do not have transportation, bus passes will be mailed to the participants’ desired location so that they can attend follow-up visits (many local shelters offer onsite mailboxes). Should high rates of missing data occur, we will employ multiple imputation methods designed for longitudinal data [64], such as R packages *mice* [99] and *pan* [100]. Other studies comparing usual care with a smartphone intervention have observed equal rates of attrition across study arms [101,102].

### Impact

Future research will refine the app for testing and dissemination to other homeless populations. Results from the Link2Care study will provide information that may be used to develop novel phone-based interventions that use EMAs to detect risky thoughts, behaviors, and situations in real time and automatically intervene (eg, calling counselors and text-based suggestions for dealing with mood or coping with stress). Future research studies will be conducted to determine the cost effectiveness of smartphone-based case management interventions, which may be lower than the cost of traditional case management or incarcerating or hospitalizing homeless adults.

## Abbreviations

EMA: ecological momentary assessment
GLMM: generalized linear mixed model
IRB: institutional review board
QDS: Questionnaire Development System
QoL: quality of life
RCT: randomized controlled trial
SPCM: smartphone-based case management
UCM: usual case management

## Appendix

Multimedia Appendix 1 Peer review summary statement.
