# Supplementary material for: Using mHealth to Increase Treatment Utilization Among Recently Incarcerated Homeless Adults (Link2Care): Protocol for a Randomized Controlled Trial
Source: JMIR Res Protoc. 2018 Jun 5;7(6):e151. doi: 10.2196/resprot.9868 (PMC6008513; doi:10.2196/resprot.9868)
Supplement: Multimedia Appendix 1 [file resprot_v7i6e151_app1.PDF]

PROGRAM CONTACT:
Adelaida Rosario
301-402-1366
hoyosar@mail.nih.gov

Release Date: 06/23/2015

Application Number: 1 R01 MD009720-01A1

Principal Investigators (Listed Alphabetically):
BUSINELLE, MICHAEL S. PHD (Contact)
REINGLE, JENNIFER M PHD

Applicant Organization: UNIVERSITY OF TEXAS HLTH SCI CTR HOUSTON

Review Group: HDEP
Health Disparities and Equity Promotion Study Section

Meeting Date: 06/11/2015
Council: OCT 2015
Requested Start: 09/01/2015
RFA/PA: PA13-302
PCC: R01
Dual IC(s): MH, DA

Project Title: mHealth to Increase Service Utilization in Recently Incarcerated Homeless Adults

SRG Action: Impact Score: 31 Percentile: 16
Next Steps: Visit http://grants.nih.gov/grants/next_steps.htm
Human Subjects: 30-Human subjects involved - Certified, no SRG concerns
Animal Subjects: 10-No live vertebrate animals involved for competing appl.
Gender: 1A-Both genders, scientifically acceptable
Minority: 1A-Minorities and non-minorities, scientifically acceptable
Children: 1A-Both Children and Adults, scientifically acceptable
Clinical Research - not NIH-defined Phase III Trial

Project Year	Direct Costs Requested	Estimated Total Cost
1	277,557	419,460
2	305,409	461,551
3	307,009	463,969
4	305,409	461,551
5	246,737	372,883
TOTAL	1,442,121	2,179,414

ADMINISTRATIVE BUDGET NOTE: The budget shown is the requested budget and has not been adjusted to reflect any recommendations made by reviewers. If an award is planned, the costs will be calculated by Institute grants management staff based on the recommendations outlined below in the COMMITTEE BUDGET RECOMMENDATIONS section.

EARLY STAGE INVESTIGATOR, NEW INVESTIGATOR

1R01MD009720-01A1 Businelle, Michael

EARLY_STAGE_INVESTIGATOR
NEW INVESTIGATOR

RESUME AND SUMMARY OF DISCUSSION: This project proposes to evaluate the impact of a smart phone intervention designed to connect recently incarcerated homeless adults to community-based case management services and resources as well as identify variables that predict continued homelessness and re-arrest. The focus on a hard to serve population with substantial health disparities and barriers to care is highly significant. The use of interactive case management through smart phones is novel, user friendly, and scalable. The investigative team has appropriate expertise, experience working with the target population, and a strong relationship with the proposed homeless shelter. The revised application is very responsive to prior review concerns. However, some weaknesses were noted. While the preliminary data supports the use of smart phones with the target population, the focus of prior work was on a different outcome (smoking) and there is insufficient discussion on how the preliminary studies support the proposed intervention. In addition, it is not clear how the intervention will be scaled up or disseminated. There was some discussion and disagreement around the inclusion of the intermediary intervention group (case management plus smart phone - UCM+SP). While some reviewers felt the group unnecessarily complicated the study with no added value, others thought the group would allow for an important assessment of the impact of the smart phone alone. Finally, some reviewers questioned the potential impact of the intervention given the intensity of problems faced by this target population. Overall, the panel agreed that the proposed study could lead to an effective, albeit modest, intervention for an underserved and hard to reach population.

DESCRIPTION (provided by applicant): There is a significant revolving door of incarceration among homeless adults, a population with substantial health disparities. Homeless adults who receive the professional coordination of individualized care (i.e., case management) during the period following their release from jail experience fewer mental health and substance use problems, are more likely to obtain stable housing, and are less likely to be re-incarcerated. This is because case managers work to meet the various needs of their clients by helping them to overcome barriers to needed services (e.g., food, clothing, housing, job training, substance abuse and mental health treatment, medical care, medication, social support, proof of identification, legal aid). Many barriers (e.g., limited transportation, inability to schedule appointments, limited knowledge of available services) prevent homeless adults who were recently released from incarceration from obtaining available case management, crisis management, substance abuse, and mental health services. The proposed study will use mobile technology to address these barriers and fill gaps in the understanding of the causes of the revolving door of homeless incarceration. Specifically, 432 homeless adults who enroll in a shelter based Homeless Recovery Program after release from county jail will be randomly assigned to one of three treatment groups: 1) usual shelter based case management (UCM), 2) UCM plus a study provided smart phone (UCM+SP), and 3) UCM with a study provided smart phone that is preloaded with an innovative care management app (SPCM). The SPCM app is an extension of the research team's previous successful work using mobile devices to assess and modify health behaviors in low income and homeless adults. Those assigned to SPCM will receive smart phones that will prompt (twice weekly) connections to shelter based case managers. The app will also offer direct links to care managers (available during normal business hours) and crisis interventionists (available 24 hours a day, 7 days a week), with the touch of a button. It is hypothesized that SPCM will increase utilization of case and crisis management services thereby addressing unmet needs (e.g., obtaining shelter, clothing, counseling, identification) and reducing homelessness and re-arrest. Another key focus of this study is to address gaps in the understanding of mechanisms that drive re-arrest and homelessness by using traditional in-person (i.e., baseline, 1, 3, and 6 months post-baseline) and smart phone based (i.e., daily for 6 months) assessment methods to identify distal and proximal predictors (e.g., affect,

thoughts, behaviors, events) of continued homelessness and arrest. This research represents a step toward integrated service connection and healthcare service provision for one of the most underserved, high need, and understudied populations in the United States. Smart phone apps that increase the use of available healthcare services and identify predictors of key outcomes (e.g., homelessness, re-arrest, medication compliance) could be used to reach hard to reach populations with histories of significant and persistent health disparities (e.g., homeless adults).

PUBLIC HEALTH RELEVANCE: Case management and crisis counseling services reduce mental health and substance abuse problems, homelessness, and re-incarceration among recently incarcerated homeless adults, a disproportionately high minority population with substantial health disparities. There are many barriers (e.g., limited transportation, inability to schedule and keep appointments, limited knowledge of available services) that prevent recently incarcerated homeless adults from obtaining these needed services and few studies have identified predictors of re-arrest and continued homelessness. The primary goals of this research are to: 1) assess the impact of an innovative smart phone application that will prompt and directly link recently incarcerated homeless adults to community-based case management services and resources, and 2) utilize in-person and smart phone based assessments to identify key variables that predict continued homelessness and re-arrest.

CRITIQUE 1

Significance: 3
Investigator(s): 1
Innovation: 1
Approach: 4
Environment: 1

Overall Impact: This is an exceptional research team taking on two of society's greatest problems simultaneously: homelessness and incarceration. The investigators comprise the epitome of the "town-gown" concept, with outstanding research productivity coupled with excellent relationships with relevant agencies in the Dallas area. The smart phone-based experiment applied to these issues makes this study unusually innovative. The approach unfortunately, even if proven feasible, could represent a mere drop in the bucket in the face of cultural prejudices, psychiatric illnesses, poverty, broken families, and other problems the target population faces. In general, the investigators have been very responsive to a previous review. Given the innovation in the approach however one questions the need for the middle arm of the 3 arm trial, as this experiment still has the feel of an efficacy trial.

1. Significance:

Strengths

- The USA is perhaps the leader among all Western democracies in terms of the proportion of homeless people, and of incarcerated and recently incarcerated adults.
- Often, homelessness is predicted by incarceration and psychiatric problems and leads to a vicious cycle that is extremely difficult to break.
- These individuals are vulnerable to an extraordinary array of physical and mental problems.

Weaknesses

- Solutions to these difficult issues are lacking and the investigators should be commended for attempting to take them on. However, it remains unclear whether this approach, while innovative, could possibly have much of an impact on such intractable problems in the context of overwhelming social and political challenges that make them so. Poor African American and other males in Dallas TX and elsewhere face extensive challenges in obtaining gainful employment, avoiding arrest, etc., whether they had previously been incarcerated or not.

2. Investigator(s):
Strengths
- Drs. Businelle and Kendzor are clinical psychologists and highly familiar with problems individuals such as those in the target population face.
- Co-Principal Investigator Dr. Reingle complements the work of Drs. Businelle and Kendzor with her background in epidemiology and the criminal justice system, highly relevant to this work as well.
- All 3 leaders are exceptionally productive.
- The MPI plan is well detailed and justified.
- This is a very strong research team.

Weaknesses
- Drs. Businelle and Kendzor overlap in their expertise and to some extent in their roles on the project.

3. Innovation:
Strengths
- The approach (both in terms of intervention and EMA) and target population are both exceptionally innovative research topics, especially in combination.

Weaknesses
- None noted.

4. Approach:
Strengths
- The overall approach is very strong in terms of population access, experimental design, sample size and data analyses, timeline and resource allocation, and other factors.
- Letters of support and access from numerous Dallas agencies document the excellent community ties that the research team has, and they should be most proud of this.
- The investigators have been responsive to the previous review of this application.

Weaknesses
- Concerns remain about the utility and feasibility of the smart phone approach, and its reliability and validity for assessing affect and related psychological issues on an EMA basis.
- Though the reapplication used additional piloting to test the receptiveness of the target population to EMA, their self-reported satisfaction of course may be subject to biases (p. 125).
- Given the innovation of the trial, one questions why the middle/intermediate arm would be needed at this juncture in the development of this research line. Perhaps one can infer this from their citation of previous programs that have already used this approach (p. 128), though this is not clear.
- The response to the previous concern about not tailoring to African Americans is not fully developed.

5. Environment:
Strengths
- The UT School of Public Health-Dallas and other settings for the investigators' work offer more than adequate support for this study.

Weaknesses
- None noted.

Protections for Human Subjects:
Acceptable Risks and/or Adequate Protections

Data and Safety Monitoring Plan (Applicable for Clinical Trials Only):
Acceptable

Inclusion of Women, Minorities and Children:
- Sex/Gender: Distribution justified scientifically
- Race/Ethnicity: Distribution justified scientifically
- Inclusion/Exclusion of Children under 21: Including ages < 21 justified scientifically

Resubmission:
- The investigators are very responsive to the previous review at least to the extent they can be without fully altering the study. Some lack of clarity persists, however, as to why African American males do not receive tailored communication.

Budget and Period of Support:
Recommend as Requested

CRITIQUE 2

Significance: 1
Investigator(s): 2
Innovation: 3
Approach: 2
Environment: 1

Overall Impact: This research application is a highly significant study that aims to reduce mental health and substance use morbidity among homeless adults being released from jail. The study uses well established case management approach, and is attempting to augment this strategy using a mobile app installed on a smartphone. The intervention team has some pilot data to support a mobile intervention but these data does not directly test the intervention proposed. If successful this approach is highly scalable and likely to be rapidly adopted in other parallel settings. The investigative team is well positioned to undertake this research and is in a supportive research environment. The Principal Investigators are both early stage investigators, and this project would be a logical starting point for future research in this domain. Weaknesses in the application appear to be minor and will likely be overcome as the project moves forward.

1. Significance:
Strengths
- The investigator makes a strong case that the “revolving door” of homelessness and incarceration results in greater mental health and substance use disorders, and risks further incarceration.
- This population has very high morbidity, and mortality given present research.
- The focus on case management makes this research potentially highly replicable at relatively low cost.

Weaknesses
- None noted.

2. Investigator(s):
Strengths
- The Principal Investigators are both Early Stage Investigators. They appear to have the requisite experience to carry out this research.

- They have addressed prior critiques and drawn in senior consultants with established research portfolios to complement their skills.

Weaknesses

- None noted.

3. Innovation:

Strengths

- The use of smartphones in a three-arm design to test the effectiveness of their mobile app is high innovative, and has the potential to rapidly translate into practice if successful.
- EMA is a very appropriate and, given the pilot data presented in the application, highly feasible technique to use in studying this population, which tends to have high mobility.
- The EMA data will be very useful in examining factors that lead to re-incarceration.

Weaknesses

- The pilot data presented are largely focused on tobacco cessation and do not address this particular case management intervention directly. This is a significant weakness.
- This is a relatively minor issue. Given the centrality of smartphones in this study consideration could have been given to having alcohol concentration monitors used in parallel to better monitor overall patterns of substance use.

4. Approach:

Strengths

- Builds on established relationships of Principal Investigator with a homeless shelter.
- Sample size considerations and power analysis appear to be well considered and thoughtfully described.
- EMA is a real strength in this study and allows for the gathering of "fine-grained" data.
- Data analysis sections are detailed, and appear appropriate to the aims of the study.

Weaknesses

- There is concern about the feasibility of this specific intervention and whether the pilot data from tobacco studies support the app-based case management approach described here.
- Consideration should be given to loss of phones (accident or sale) – will the investigators replace the phone and for what period? Will they be able to remotely erase or "kill" a lost phone?

5. Environment:

Strengths

- The research team appears to have appropriate resources and support to carry out this study.
- Consultants from the University of Kentucky and Pacific Institute for Research & Evaluation add substantively to the application.
- Solid collaborations with Bridge Steps (adult homeless shelter) and with Dallas County Jail.

Weaknesses

- None noted.

<u>Protections for Human Subjects:</u>

Acceptable Risks and/or Adequate Protections

Data and Safety Monitoring Plan (Applicable for Clinical Trials Only):

Acceptable

- Given the minimal risk of this application, DSMP is appropriate to have monitoring by Principal Investigators.

<u>Inclusion of Women, Minorities and Children:</u>

- Sex/Gender: Distribution justified scientifically
- Race/Ethnicity: Distribution justified scientifically
- Inclusion/Exclusion of Children under 21: Including ages < 21 justified scientifically
- The application will recruit eligible subjects aged 18-21 who are considered children under NIH policy.

Resubmission:
- The investigators have substantively responded to critiques from the prior review, and have made changes that strengthen the application.

Budget and Period of Support:
Recommend as Requested

CRITIQUE 3

Significance: 1
Investigator(s): 1
Innovation: 1
Approach: 1
Environment: 1

Overall Impact: The project is likely to have sustained, high impact given the technological advances of smart phone and smart phone enhanced prompts to positive help-seeking behaviors among a high-risk population of recently incarcerated male and female adults. The relatively low cost of smart phone technology outweighs the personal and societal costs due to re-arrest and incarceration. Identifying if the intervention has similar effects across race, gender, and age will advance the field of health disparities particularly with respect to potentially modifiable areas for future service provision. There is evidence that the collaborators have had strong prior relationships with the recruitment/service sites, and have a clear theoretical framework for changing help-seeking behaviors in adults with a myriad of barriers to service receipt and retention.

1. Significance:
Strengths
- The project is likely to advance the field by applying innovative technology that is useable, scalable across “real world” settings. Now that smart phone use is more commonplace, even in minority communities, the results of the project are likely to be of significant consequence to persons and care systems.

Weaknesses
- None noted.

2. Investigator(s):
Strengths
- The team of investigators is strong, and has had much experience in technology and service use.
- The combination of investigators and expertise is much more enhanced by the incorporation of a consultant with substantial case management research experience.

Weaknesses
- None noted.

3. Innovation:
Strengths

- The use of smart phones as mHealth technology is the innovation.
- This innovation is buttressed by the 3-arm randomized behavioral trial design, which may show a gradient of improvements in the major variables of interest.
- Adding data collection on key proximal and distal factors influencing re-arrest/incarceration/homelessness is a keen way to identify intervention targets in the future.

Weaknesses

- None noted.

4. Approach:
Strengths

- The investigators were responsive to decreasing the degree of respondent burden that could emerge from time-invasive experimental procedures such as the repeated, daily EMAs (ecological momentary assessments).
- The design is comprehensive and coheres with the stated specific aims.
- The analytic approach is sound, and the investigators have prior experience in conducting this type of mHealth studies.
- The investigators identify that only .8 percent of clients in the Bridge Homeless Recovery Program were non-English speakers. This decreases the need at this time to adapt the interventions for non-English speakers.

Weaknesses

- None noted.

5. Environment:
Strengths

- The environment is adequate and the equipment needed for the project is accessible and user friendly.

Weaknesses

- None noted.

Protections for Human Subjects:
Acceptable Risks and/or Adequate Protections
Data and Safety Monitoring Plan (Applicable for Clinical Trials Only):
Not Applicable (No Clinical Trials)

Inclusion of Women, Minorities and Children:

- Sex/Gender: Distribution justified scientifically
- Race/Ethnicity: Distribution justified scientifically
- Inclusion/Exclusion of Children under 21: Excluding ages < 21 justified scientifically

Resubmission:

- The investigators responded adequately to the prior reviewer critiques.

Budget and Period of Support:
Recommend as Requested

THE FOLLOWING SECTIONS WERE PREPARED BY THE SCIENTIFIC REVIEW OFFICER TO SUMMARIZE THE OUTCOME OF DISCUSSIONS OF THE REVIEW COMMITTEE, OR REVIEWERS' WRITTEN CRITIQUES, ON THE FOLLOWING ISSUES:

PROTECTION OF HUMAN SUBJECTS (Resume): ACCEPTABLE

INCLUSION OF WOMEN PLAN (Resume): ACCEPTABLE

INCLUSION OF MINORITIES PLAN (Resume): ACCEPTABLE

INCLUSION OF CHILDREN PLAN (Resume): ACCEPTABLE

COMMITTEE BUDGET RECOMMENDATIONS: The budget was recommended as requested.

NIH has modified its policy regarding the receipt of resubmissions (amended applications). See Guide Notice NOT-OD-14-074 at http://grants.nih.gov/grants/guide/notice-files/NOT-OD-14-074.html. The impact/priority score is calculated after discussion of an application by averaging the overall scores (1-9) given by all voting reviewers on the committee and multiplying by 10. The criterion scores are submitted prior to the meeting by the individual reviewers assigned to an application, and are not discussed specifically at the review meeting or calculated into the overall impact score. Some applications also receive a percentile ranking. For details on the review process, see http://grants.nih.gov/grants/peer_review_process.htm#scoring.

MEETING ROSTER

Health Disparities and Equity Promotion Study Section
Healthcare Delivery and Methodologies Integrated Review Group
CENTER FOR SCIENTIFIC REVIEW
HDEP
June 11, 2015 - June 12, 2015

CHAIRPERSON

SOMMERS, MARILYN S, RN, PHD
LILLIAN S. BRUNNER PROFESSOR OF MEDICAL SURGICAL NURSING
SCHOOL OF NURSING
UNIVERSITY OF PENNSYLVANIA
PHILADELPHIA, PA 19104

MEMBERS

AJROUCH, KRISTINE J, PHD *
ADJUNCT RESEARCH PROFESSOR
INSTITUTE FOR SOCIAL RESEARCH
UNIVERSITY OF MICHIGAN
ANN ARBOR, MI 48106

ANDERSON, ROGER T, PHD *
PROFESSOR, ASSOCIATE DIRECTOR
CO-LEADER FOR CANCER CONTROL AND POPULATION HEALTH
DEPARTMENT OF PUBLIC HEALTH
UNIVERSITY OF VIRGINIA SCHOOL OF MEDICINE
CHARLOTTESVILLE, VA 22903

ANTHONY, RENAISA SPRING MDMPH, MPH, MD *
DEPUTY DIRECTOR/ASSISTANT PROFESSOR
CENTER FOR REDUCING HEALTH DISPARITIES
UNIVERSITY OF NEBRASKA MEDICAL CENTER
OMAHA, NE 68198

ARANDA, MARIA P., PHD *
ASSOCIATE PROFESSOR
SCHOOL OF SOCIAL WORK
UNIVERSITY OF SOUTHERN CALIFORNIA
LOS ANGELES, CA 90089

BERRY, CAROLYN ANNE, PHD
ASSOCIATE PROFESSOR
DEPARTMENT OF POPULATION HEALTH
NEW YORK UNIVERSITY SCHOOL OF MEDICINE
NEW YORK, NY 10016

BOUTIN-FOSTER, CARLA , MD *
ASSOCIATE PROFESSOR
WEI II CORNELL MEDICAL COLLEGE
NEW YORK, NY 10021

CABASSA, LEOPOLDO J., PHD *
ASSOCIATE PROFESSOR
COLUMBIA UNIVERSITY
SCHOOL OF SOCIAL WORK
NEW YORK, NY 10032

CARDARELLI, KATHRYN MARIE, PHD *
ASSOCIATE DEAN FOR ACADEMIC AND STUDENT AFFAIRS
DEPARTMENT OF HEALTH BEHAVIOR
UNIVERSITY OF KENTUCKY
COLLEGE OF PUBLIC HEALTH
LEXINGTON, KY 40536

ELDER, JOHN P, PHD
PROFESSOR OF PUBLIC HEALTH
GRADUATE SCHOOL OF PUBLIC HEALTH
SAN DIEGO STATE UNIVERSITY
SAN DIEGO, CA 92123

FERNANDER, ANITA F, PHD *
ASSOCIATE PROFESSOR, DIRECTOR OF GRADUATE STUDIES
DEPARTMENT OF BEHAVIORAL SCIENCE
COLLEGE OF MEDICINE
UNIVERSITY OF KENTUCKY
LEXINGTON, KY 40536

FREUND, KAREN, MD,MPH , MD *
PROFESSOR AND VICE CHAIR OF MEDICINE
TUFTS MEDICAL CENTER
TUFTS UNIVERSITY
BOSTON, MA 02111

GARCIA, VICTOR Q, PHD *
PROFESSOR OF ANTHROPOLOGY & DIRECTOR
MID-ATLANTIC RESEARCH AND
TRAINING INSTITUTE (MARTI)
INDIANA UNIVERSITY OF PENNSYLVANIA
INDIANA, PA 15701

HATZENBUEHLER, MARK LOUIS, PHD *
ASSISTANT PROFESSOR
DEPARTMENT OF SOCIOMEDICAL SCIENCES
COLUMBIA UNIVERSITY
NEW YORK CITY, NY 10032

JANDORF, LINA , MA *
RESEARCH PROFESSOR
DEPARTMENT OF ONCOLOGICAL SERVICES
ICAHN SCHOOL OF MEDICINE AT MOUNT SINAI
NEW YORK CITY, NY 10029

KARNIK, NIRANJAN , MD, PHD *
ASSOCIATE PROFESSOR
DEPARTMENT PSYCHIATRY
RUSH UNIVERSITY MEDICAL CENTER
CHICAGO, IL 60612

LENGERICH, EUGENE J, VMD *
PROFESSOR
DEPARTMENT OF PUBLIC HEALTH SCIENCES
PENNSYLVANIA STATE UNIVERSITY
HERSHEY, PA 17033

MARTINEZ, CHARLES R JR, PHD
PROFESSOR AND DIRECTOR
CENTER FOR EQUITY PROMOTION
DEPARTMENT OF EDUCATIONAL METHODOLGY, POLICY AND LEADERSHIP
UNIVERSITY OF OREGON
EUGENE, OR 97403

MENON, USHA , RN, PHD, FAAN
CENTENIAL PROFESSOR OF NURSING, DIRECTOR
COMMUNITY ENGAGEMENT CCTS
COLLEGE OF NURSING
THE OHIO STATE UNIVERSITY
COLUMBUS, OH 43210

MUNOZ-LABOY, MIGUEL A, DPH *
ASSOCIATE PROFESSOR
SCHOOL OF SOCIAL WORK
TEMPLE UNIVERSITY
PHILADELPHIA, PA 19122

NUNEZ-SMITH, MARCELLA , MD *
ASSOCIATE PROFESSOR OF MEDICINE AND PUBLIC HEALTH
EQUITY RESEARCH AND INNOVATION CENTER
YALE UNIVERSITY
NEW HAVEN, CT 06520

NUNN, MARTHA E, PHD, DDS *
ASSOCIATE PROFESSOR AND DIRECTOR
CENTER FOR ORAL HEALTH RESEARCH
DEPARTMENT OF PERIODONTOLOGY
SCHOOL OF DENTISTRY
CREIGHTON UNIVERSITY
OMAHA, NE 68178

OATES, JAMES C, MD *
ASSOCIATE PROFESSOR
DEPARTMENT OF MEDICINE
MEDICAL UNIVERSITY OF SOUTH CAROLINA
CHARLESTON, SC 29425

ORTEGA, ALEXANDER N, PHD
PROFESSOR
UCLA CENTER FOR POPULATION HEALTH
AND HEALTH DISPARITIES
SCHOOL OF PUBLIC HEALTH
UNIVERSITY OF CALIFORNIA, LOS ANGELES
LOS ANGELES, CA 90095

PEARMAN, TIMOTHY PATRICK, PHD *
ASSOCIATE PROFESSOR, DIRECTOR SUPPORTIVE ONCLOLOGY
LURIE COMPREHENSIVE CANCER CENTER
NORTHWESTERN UNIVERSITY SCHOOL OF MEDICINE
CHICAGO, IL 60611

POLIVKA, BARBARA J, PHD *
SHIRLEY B. POWERS ENDOWED CHAIR AND PROFESSOR
UNIVERSITY OF LOUISVILLE SCHOOL OF NURSING
NORTON HEALTHCARE
LOUISVILLE, KY 40202

POSTON, WALKER S., PHD *
DEPUTY DIRECTOR AND SENIOR PRINCIPAL INVESTIGATOR
INSTITUTE FOR BIOBEHAVIORAL HEALTH RESEARCH
CENTER FOR FIRE, RESCUE & EMS HEALTH RESEARCH
NATIONAL DEVELOPMENT AND RESEARCH INSTITUTES NDRI
NEW YORK, NY 10010

PREWITT, T ELAINE, DRPH *
ASSOCIATE PROFESSOR AND DEPUTY DIRECTOR
DEPARTMENT OF HEALTH POLICY AND MANAGEMENT
FAY W. BOOZMAN COLLEGE OF PUBLIC HEALTH
UNIVERSITY OF ARKANSAS FOR MEDICAL SCIENCES
LITTLE ROCK, AR 72205

RADECKI BREITKOPF, CARMEN , PHD *
ASSOCIATE PROFESSOR
DIVISION OF HEALTH CARE POLICY AND RESEARCH
DEPARTMENT OF HEALTH SCIENCES RESEARCH
MAYO CLINIC COLLEGE OF MEDICINE
ROCHESTER, MN 55905

SANDERS-PHILLIPS, KATHY , PHD *
SENIOR BEHVIORAL SCIENTIST
DEPARTMENT OF PEDIATRICS AND CHILD HEALTH
COLLEGE OF MEDICINE
HOWARD UNIVERSITY
WASHINGTON, DC 20001

SCHOLD, JESSE D, PHD *
ASSOCIATE STAFF, DIRECTOR OF OUTCOMES
RESEARCH AND MEDICAL INFORMATICS
DEPARTMENT OF QUANTITATIVE HEALTH SCIENCES
LERNER RESEARCH INSTITUTE
CLEVELAND CLINIC
CLEVELAND, OH 44195

SHALOWITZ, MADELEINE ULLMAN, MD
DIRECTOR OF BIOMEDICAL RESEARCH AND RESEARCH ASSOCIATE
DEPARTMENT OF BIOMEDICAL RESEARCH
UNIVERSITY OF CHICAGO
EVANSTON, IL 60201

SHUMWAY, MARTHA , PHD *
ASSOCIATE PROFESSOR
DEPARTMENT OF PSYCHIATRY
UNIVERSITY OF CALIFORNIA SAN FRANCISCO
BOX 0884
SAN FRANCISCO, CA 94143

VIDRINE, JENNIFER IRVIN, PHD *
DEPUTY DIRECTOR FOR TOBACCO RESEARCH AND DIRECTOR
STEPHENSON CANCER CENTER
ASSOCIATE PROFESSOR, DEPARTMENT OF FAMILY
AND PREVENTIVE MEDICINE
UNIVERSITY OF OKLAHOMA HEALTH SCIENCES CENTER
OKLAHOMA CITY, OK 73104

WU, BEI , PHD *
PAULINE GRATZ PROFESSOR OF NURSING AND
PROFESSOR OF GLOBAL HEALTH
DIRECTOR FOR INTERNATIONAL RESEARCH, SCHOOL OF NUR
DUKE UNIVERSITY
DURHAM, NC 27710

SCIENTIFIC REVIEW OFFICER

OLUFOKUNBI SAM, DELIA , PHD
SCIENTIFIC REVIEW OFFICER
CENTER FOR SCIENTIFIC REVIEW
NATIONAL INSTITUTES OF HEALTH
BETHESDA, MD 20892

EXTRAMURAL SUPPORT ASSISTANT

AKINLOLU, ABDUL JABBAR O BA
LEAD EXTRAMURAL SUPPORT SPECIALIST
CENTER FOR SCIENTIFIC REVIEW
NATIONAL INSTITUTES OF HEALTH
BETHESDA, MD 20892

* Temporary Member. For grant applications, temporary members may participate in the entire meeting or may review only selected applications as needed.

Consultants are required to absent themselves from the room during the review of any application if their presence would constitute or appear to constitute a conflict of interest.
